# Prioritization of Candidates for HTA: Insights from Indian Healthcare Landscape

**DOI:** 10.1136/bmjebm-2023-112566

**Published:** 2025-11-26

**Authors:** Malkeet Singh, Abha Mehndiratta, Manuel Antonio Espinoza, Shankar Prinja, Ursula Giedion

**Affiliations:** Center for Global Development, Washington, DC, US; Center for Global Development, Washington DC Washington, US 20036-1915; Center for Global Development, Washington, DC, US; PGIMER, Chandigarh, Chandigarh, IN; Center for Global Development, Washington, DC, US

**Keywords:** Public Health, Health Care Quality, Access, and Evaluation, Health Services Research

## Background

Health Technology Assessment (HTA) is a multidisciplinary process that employs explicit methods to evaluate the value of a health technology. This evaluation encompasses various dimensions of value, which are assessed by examining both the intended and unintended consequences of using the technology in comparison to existing alternatives. These dimensions typically include clinical effectiveness, safety, costs and economic implications, and extend to ethical, social, cultural, and legal issues, as well as organizational and environmental aspects. Additionally, HTA considers broader implications for patients, their relatives and caregivers, and the population as a whole.^1^ The overall value of a health technology can vary depending on the perspective adopted, the stakeholders engaged, and the specific context of the decision-making process.

In India, the Health Technology Assessment India (HTAIn), which is a recently established HTA body of the country, has made significant progress over the past seven years in embedding HTA into the healthcare system to support evidence-based decision-making. A recent study demonstrated that investments in HTAIn yielded returns ranging from 5:1 to 40:1, depending on the specific health interventions evaluated. ^2^ This underscores the importance of carefully selecting priority topics for assessment to maximize the impact of HTA in enhancing healthcare resource allocation and achieving better health outcomes.

HTA systems operate within the constraints of finite resources for conducting assessments. Consequently, not all potential candidates for inclusion can undergo a full HTA. Prioritizing technologies for evaluation is therefore critical, focusing on those with the potential to provide most value. Additionally, not all candidates necessitate a comprehensive evaluation; in cases where there is a high confidence regarding their inclusion or exclusion, decisions can often be informed by rapid review of existing literature or real-world data.^3^

Topic prioritization is a cornerstone of optimizing the use of limited HTA resources, aiming to maximize their contribution to strengthening the healthcare system. This process involves identifying technologies most likely to benefit from in-depth HTA evaluation. To ensure fairness and transparency, topic prioritization must follow an evidence-based, explicit, and well-defined framework. Simultaneously, it must remain pragmatic, aligning with the country’s health system priorities and striking a balance between methodological rigor and practical feasibility.

## Framework for Prioritization of Topics for HTA in India

Topic prioritization for HTA is often perceived as a process that occurs solely at the level of one, national, HTA body.^4^ However, in this commentary, we illustrate how topic prioritization in India operates at multiple levels and how its objectives and methodologies vary depending on who is in charge and which stakeholders are involved. The filtering of topics takes place at distinct levels: first, at the level of publicly funded bodies (referred to as HTA “user” departments) that are authorized to propose topics for assessment to HTAIn, and second, at the level of HTAIn itself, which further prioritizes the topics submitted by these public entities (see supplementary file).

India’s pluralistic health system comprises multiple public and private sector budget holders operating at both national and State levels. Within this framework, only publicly funded bodies at the national and State levels are authorized to submit topics for HTA.^5^ At the first level of topic prioritization, various government stakeholders identify potential HTA candidates based on nominations received through advocacy from various sources, including physicians, industry representatives, patients, and civil society. Additionally, candidates are identified through horizon scanning, which involves systematically reviewing local and international literature to identify innovations relevant and potentially beneficial in the Indian context.^6^ While most topics focus on the adoption of new technologies, some also involve disinvestment strategies, reassessing existing technologies to modify their use, intensity, or scope of application^6^. The prioritization process at this first level typically uses criteria such as clinical effectiveness, policy relevance, and budget impact—though it maybe applied in a flexible and non-systematic manner—and is designed to ensure that HTA candidates align with the goals and mandates of the respective department. For instance, the National Health Mission may prioritize technologies that address maternal and child health or non-communicable diseases, which are key focus areas of the mission, while the National Program for Elimination of Tuberculosis will focus on interventions that enhance diagnosis, treatment, or prevention of tuberculosis to achieve its eradication targets. Similarly, the National Health Authority (NHA), the agency responsible for designing and implementing India’s public health insurance programs, will prioritize topics that improve financial risk protection, expand access to high-quality care for beneficiaries, or enhance the efficiency of the Health Benefits Package under Ayushman Bharat Pradhan Mantri Jan Arogya Yojana (PM-JAY), the country’s flagship scheme providing free hospitalization coverage to low-income families^7^. This decentralized, multi-level topic prioritization process ensures that nominated technologies are not only relevant to the overarching goals of the health system but also directly contribute to the distinct objectives and priorities of the respective public programs and their stakeholders who use HTA to inform their decisions.

The criteria employed by HTA “user” departments to filter and prioritize technologies for evaluation by HTAIn, and to determine whether a full HTA is warranted, or whether other, more rapid evidence based methods of recommending inclusions or exclusions may be sufficient, vary significantly. Given that a full HTA typically takes 12 to 18 months, decisions regarding the funding of technologies identified by user departments cannot always wait for its completion. This lengthy turnaround time risks delaying decisions that directly impact patient access to essential healthcare services. To address this challenge, this first-level de-prioritization process is employed, making sure scarce HTA resources focus on topics requiring comprehensive evaluation. Technologies not prioritized for a full HTA at this stage are not automatically included or excluded from policy consideration. Instead, decisions for such candidates may be guided by available evidence and traditional processes, such as expert opinion and stakeholder engagement. For instance, technologies that clearly constitute essential services, such as cesarean deliveries for complicated pregnancies, or those falling under exclusion criteria, such as aesthetic augmentation procedures, are typically addressed without a full HTA. Similarly, technologies lacking adequate evidence on clinical effectiveness or safety are deprioritized for HTA evaluation until better evidence is available. When local or international HTA evidence provides consistent and robust evidence to demonstrate, either clear cost-inefficiency or cost savings, rapid decision-making may be pursued after appropriate contextualization, especially for time-sensitive topics with low budgetary impact. This approach, termed Adaptive HTA (aHTA), reduces duplication of efforts, expedites decision-making, and preserves resources for evaluating technologies with greater uncertainty or substantial budget implications^3^. By integrating aHTA, the process becomes more efficient, agile and responsive to the immediate needs of the healthcare system.

While the prioritization process at the first level is often ad hoc using implicit criteria some departments at this level, such as the NHA, have implemented standardized policies and explicit criteria to streamline candidate selection (see supplementary file). The NHA, has established the Health Financing and Technology Assessment (HeFTA) unit, with one of its key functions being the prioritization of topics for HTA^6^. The unit selects topics from stakeholder nominations submitted via a dedicated NHA portal and conducts horizon scanning to identify and evaluate emerging HTA evidence relevant to PM-JAY beneficiaries. The prioritization process is guided by a structured set of criteria, including the burden of disease, budget and economic impact, feasibility, clinical impact, availability of quality evidence, health policy relevance, and social value judgments. This systematic approach ensures that HTA efforts are directed toward high-impact interventions aligned with the goals of the PM-JAY scheme.

Topics selected by user departments undergo further scrutiny through a second filter at HTAIn, India’s Health Technology Assessment body, which is exclusively government-funded. At present, the HTAIn secretariat, in collaboration with the HTAIn Technical Appraisal Committee (HTAIn-TAC), prioritizes interventions that are of national importance and have the potential to deliver substantial health benefits. While it currently evaluates only those interventions prioritized by publicly funded bodies, it will soon accept nominations from the private sector. Accepting submissions from the private sector will introduce new challenges, including higher demand for evaluations and increased industry pressure to prioritize topics that may not align with public health priorities, such as essential medicines. Instead, there may be a bias toward high-cost, emerging technologies like patented oncology drugs and advanced diagnostic tests. To address these, HTAIn must strengthen its topic selection process to balance industry demands with community health needs, while ensuring scientific rigor, transparency and objectivity. Additionally, its capacity to conduct HTA activities will need to be reinforced to manage the growing workload effectively.

HTAIn employs a two-stage process for it topic prioritization. In the first stage, two key criteria are evaluated: whether the research topic aligns with HTAIn’s mandate and qualifies for an HTA study, and the regulatory approval status of the healthcare technology. The second stage involves a more comprehensive assessment by the HTAIn Topic Prioritization Committee, where several criteria are scored on a scale from 1 to 5. These include the size of population affected by the disease, burden of disease (in terms of prevalence and severity), clinical impact (the potential to improve patient outcomes), economic impact (the effect on household expenditure), importance of the health intervention for policy objectives, and the availability of supporting evidence, such as RCTs, systematic reviews, or feasibility studies for conducting HTA.^5^ The candidates that receive a high score undergo further examination by the HTAIn-TAC. Based on the assessment, a decision is made regarding whether the candidate should proceed to a full HTA. In cases where more data is anticipated from ongoing studies or the intervention is deemed lower priority, the HTA may be postponed and placed on a ‘waiting list.’ Alternatively, the HTA for some interventions may be rejected due to their comparatively lower priority.

## Strengthening Topic Prioritization for HTA in India: Challenges and Opportunities

While the country’s multi-level prioritization framework has been instrumental in streamlining the HTA process, opportunities for enhancement remain. These include improving stakeholder awareness, fostering better coordination, adopting explicit timelines, and minimizing operational delays in topic prioritization at HTAIn and other State- and Center-level stakeholders, such as the NHA. Strengthening these areas would improve responsiveness to submissions and boost stakeholder confidence and system credibility.

Future refinements could focus on expanding the use of systematic first-level filtering processes across all public bodies, modeled on the NHA’s approach, to ensure uniformity and effectiveness. Strengthening the prioritization process at the State level, under the State health departments, would also be pivotal. This could involve integrating inputs from key stakeholders such as State Health Agencies, State Health Societies, Directorate of Health Services, State Medical Corporations, tertiary care institutions, and district health offices. Additionally, simplifying and refining prioritization criteria to make them measurable, non-redundant, and distinct would enhance the robustness of the process. Each criterion should uniquely capture a specific dimension of value, ensuring mutual exclusivity and avoiding overlap, while remaining measurable to facilitate practical application. Finally, periodic evaluation of the topic prioritization process will help identify challenges and uncover opportunities for optimization. By incorporating these improvements, India’s HTA prioritization framework can become more efficient and better aligned with healthcare system goals, ensuring that HTA resources are directed where they are needed most.

## Conclusion

Topic prioritization for HTA in India takes place at multiple levels. This decentralized approach serves as a critical mechanism to filter and streamline topics for assessment, enhancing the efficiency of the HTA process by ensuring that only the most relevant and impactful technologies are selected for full evaluation. For HTA user departments, prioritizing candidates for HTA is essential to align with their specific goals and mandates, particularly when there is significant uncertainty regarding a technology’s value or when the expected budgetary impact is substantial. Similarly, HTAIn, which receives topics from various user departments, carefully prioritizes interventions to address the most pressing health needs of the Indian population.

Looking ahead, the planned opening of HTA topic nominations to the private sector presents both opportunities and challenges. Expanding the pool of potential topics could improve the comprehensiveness of the HTA agenda by incorporating innovations and technologies emerging from private industry. However, this also underscores the need for a robust and transparent prioritization framework to manage competing interests and maintain alignment with public health priorities. Strengthening stakeholder engagement, establishing explicit timelines, and ensuring transparent evaluation of both public and private sector submissions will be essential to maintaining the credibility and effectiveness of India’s HTA ecosystem.

## Supplementary Material

Supp1
