# Supplementary material for: Prioritization of Candidates for HTA: Insights from Indian Healthcare Landscape
Source: BMJ Evid Based Med. Author manuscript; Available in PMC 2025 Aug 16. (PMC12355834; doi:10.1136/bmjebm-2023-112566)
Supplement: Supp1 [file NIHMS2079969-supplement-Supp1.docx]

**Supplementary Material**

HTAIn Topic Prioritisation Criteria:

| Indicator | Key question to be addressed |
| --- | --- |
| Size of population affected disease | How many people are affected by the disease or health problem that is treated or prevented by the proposed intervention among Indian population at a specified time? |
| Severity of disease/health problems | What is the severity of disease or health problem that is treated or prevented by the proposed intervention by considering the burden of disease/health problems? |
| Comparative effectiveness of health intervention | How good is the intervention at doing what it is supposed to do (e.g. a drug to treat pain, a BP device to diagnose hypertension, or a vaccine to prevent rotavirus) compared to what is already being done at present? |
| Inequality, ethics, and equity | What is the situation of inequality in accessibility to and utilization of health services by region and population? Are the poorer and more vulnerable sections facing greater difficulty in access and utilization? Are there important ethical and equity considerations in relation to the intervention in question? |
| Economic impact on household expenditure | What is the impact on household expenditure as a consequence of providing the health intervention to a family member. Does it lead to catastrophic health expenditure and impoverishment due to healthcare costs? |
| Availability and relevance of evidence | What is the availability and relevance of evidence required for conducting HTA? |
| Health sector priority and policy objective | Is the clinical area of interest a well-recognised local and/or national priority? |
| Feasibility of implementation | Can the proposed intervention be implemented in reality within what are the constraints of the Indian health system? |

Reference: Department of Health Research, Ministry of Health and Family Welfare, Government of India. Health technology assessment in India: a manual. New Delhi: Department of Health Research, 2018

NHA Topic Prioritisation Criteria:

| **Criteria** | **Details** |
| --- | --- |
| **Check for eligibility** | Eligible:   - All types of health technologies including drugs, medical devices, surgical interventions, are eligible for HTA   Not Eligible:   - Food and nutritional supplements; - Cosmetics; - Unlicensed drugs; - Technologies that are still in the research stage of development; - Models of care (the way health services are delivered, which outlines best practice of care and services for the patient cohort as they progress through the stages of a condition); - IT systems (i.e., a software platform for pre-operative surgery planning); - Telemedicine; - Population screening tests; - Cell and tissue therapy (under a different work stream) |
| **Duplication check** | Check if an HTA is already done on the topic in India- existing HTAIn reports, existing published Indian economic evaluations |
| **Magnitude of health problem/Burden of disease** | Disease/condition impacting a larger proportion of population |
| **Budget/ Economic Impact** | High costs for healthcare system due to common use (disease burden) or high unit costs,  High associated costs to patients (out-of-pocket expenditure)/ catastrophic healthcare expenditure |
| **Feasibility** | The healthcare system capacity to introduce the new intervention as routine care. The new technology does not require additional infrastructure or human resource or the additional infrastructure/ human resource/skills required can be provided for within the existing systems |
| **Clinical Impact** | The technology should have clear health benefits significantly greater than the current treatment |
| **Availability of quality evidence** | Existence of quality data (preferably from India) on the clinical effectiveness of the technology under consideration as compared to the existing intervention |
| **Health policy need** | Represents important uncertainty in decision making: variability in clinical practice, variability in recommendation in various guidelines. If there are limited comparators for indication then the prioritization increases due to greater need for approved treatments. If disease prevalence is very high then there might be a greater for the new treatment to become available |
| **Social value judgement** | Potential ethical or equity implications |

**Reference:**

National Health Authority, Ministry of Health and Family Welfare, Government of India. Provider Payments and Price Setting under PM-JAY, Improving Efficiency, Acceptability, Quality & Sustainability: Policy Document. New Delhi, National Health Authority, 2022
